# Attenuation of microRNA-16 derepresses the cyclins D1, D2 and E1 to provoke cardiomyocyte hypertrophy

**DOI:** 10.1111/jcmm.12445

**Published:** 2015-01-13

**Authors:** Shuai Huang, Xiao Zou, Jie-Ning Zhu, Yong-Heng Fu, Qiu-Xiong Lin, Ye-You Liang, Chun-Yu Deng, Su-Juan Kuang, Meng-Zhen Zhang, Yu-Lin Liao, Xi-Long Zheng, Xi-Yong Yu, Zhi-Xin Shan

**Affiliations:** a Medical Research Department of Guangdong General Hospital, Guangdong Provincial Cardiovascular Institute, Guangdong Academy of Medical SciencesGuangzhou, China; b School of Pharmaceutical Sciences, Southern Medical UniversityGuangzhou, China; c School of Basic Medical Sciences, Southern Medical UniversityGuangzhou, China; d The Libin Cardiovascular Institute of Alberta, Department of Biochemistry & Molecular Biology, The University of CalgaryCalgary, AB, Canada

**Keywords:** microRNA-16, cardiac hypertrophy, cardiomyocyte, cyclin, retinoblastoma protein

## Abstract

Cyclins/retinoblastoma protein (pRb) pathway participates in cardiomyocyte hypertrophy. MicroRNAs (miRNAs), the endogenous small non-coding RNAs, were recognized to play significant roles in cardiac hypertrophy. But, it remains unknown whether cyclin/Rb pathway is modulated by miRNAs during cardiac hypertrophy. This study investigates the potential role of microRNA-16 (miR-16) in modulating cyclin/Rb pathway during cardiomyocyte hypertrophy. An animal model of hypertrophy was established in a rat with abdominal aortic constriction (AAC), and in a mouse with transverse aortic constriction (TAC) and in a mouse with subcutaneous injection of phenylephrine (PE) respectively. In addition, a cell model of hypertrophy was also achieved based on PE-promoted neonatal rat ventricular cardiomyocyte and based on Ang-II-induced neonatal mouse ventricular cardiomyocyte respectively. We demonstrated that miR-16 expression was markedly decreased in hypertrophic myocardium and hypertrophic cardiomyocytes in rats and mice. Overexpression of miR-16 suppressed rat cardiac hypertrophy and hypertrophic phenotype of cultured cardiomyocytes, and inhibition of miR-16 induced a hypertrophic phenotype in cardiomyocytes. Expressions of cyclins D1, D2 and E1, and the phosphorylated pRb were increased in hypertrophic myocardium and hypertrophic cardiomyocytes, but could be reversed by enforced expression of miR-16. Cyclins D1, D2 and E1, not pRb, were further validated to be modulated post-transcriptionally by miR-16. In addition, the signal transducer and activator of transcription-3 and c-Myc were activated during myocardial hypertrophy, and inhibitions of them prevented miR-16 attenuation. Therefore, attenuation of miR-16 provoke cardiomyocyte hypertrophy *via* derepressing the cyclins D1, D2 and E1, and activating cyclin/Rb pathway, revealing that miR-16 might be a target to manage cardiac hypertrophy.

## Introduction

Cardiac hypertrophy can be a compensatory adaptation in response to a number of stimuli including mechanical stimulus, hormones, cytokines, growth factors or pressure overload, but pathological cardiac hypertrophy may lead to cardiomyopathy and heart failure 1. Different signal pathways are involved in physiological hypertrophy or pathological hypertrophy by different stimuli 2. Accumulating studies reveal that pathological hypertrophy can be resulted from activations of the following signal pathways, including β-adrenergic receptor-cAMP-PKA, GPCR-Gaq/PLC-PKCα, Ca^2+^/calmodulin-dependent kinase II and small G proteins-MAPK signalling cascade 3. Recent evidence indicate that Cyclin D (CCND) and cyclin D kinases (CDKs) are up-regulated by hypertrophic stimuli, and cyclin/Rb pathway participates in cardiac hypertrophy 4–10. However, the mechanism underlying modulation of cell cycle regulatory proteins and activation of cyclin/Rb pathway is not well understood.

MicroRNAs (miRs) are endogenous 20–23-nucleotide non-coding RNAs that negatively regulate gene expression at the post-transcriptional level by degrading or deadenylating the target mRNAs or by inhibiting their translation 11. miRs have been reported to play important roles in pathological cardiac hypertrophy 12–19. However, it remains largely unknown whether miRs participate in cardiac hypertrophy through modulating cyclins and cyclin/Rb signal pathway.

In this study, it has been suggested that microRNA-16 (miR-16) modulates the cell cycle regulatory proteins and cyclin/Rb pathway during cardiomyocyte hypertrophy. Our data have shown that miR-16 is down-regulated in hypertrophic myocardium and cardiomyocytes. As expected, overexpression of miR-16 can ameliorate the hypertrophic phenotype *in vitro* and *in vivo* through targeting the expression of CCND1, CCND2 and CCNE1. We have also demonstrated that the pathway involving STAT3/c-Myc activation mediates down-regulation of miR-16 in hypertrophic cardiomyocytes.

## Materials and methods

### Vector and reagents

DNA template for miR-16 precursor was amplified from rat genomic DNA using PCR technique. Recombinant adenovirus vector expressing miR-16 was generated by cloning miR-16 DNA template into pAdTrack-CMV (Stratagene, La Jolla, CA, USA), which was further recombined with pAdEasy-I (Stratagene) for the construction of recombinant miR-16 adenovirus in human embryonic kidney 293 cells. Neonatal rat ventricular cells (NRVCs) were maintained in DMEM supplemented with 10% foetal bovine serum (FBS). PE, fluorescein isothiocyanate (FITC)-phalloidin, Hoechst 33258, 10058-F4 and cryptotanshinone were purchased from Sigma-Aldrich (St. Louis., MO, USA). miR-16 mimic and inhibitor, siRNAs for CCND1, CCND2, CCNE1 and the cell-light™ EU detection kit were provided by RiboBio (Guangzhou, China).

### Animal studies

Male Sprague–Dawley (SD) rats (180–240 g), and male C57BL6 mice (20–25 g) were purchased from Laboratory Animal Center of Sun Yat-sen University. All animals were housed under pathogen-free conditions and kept on standard mouse chow with free access to tap water. This study conformed to the Guide for the Care and Use of Laboratory Animals published by the US National Institutes of Health (8th Edition, National Research Council, 2011). The present programme was also approved by the research ethics committee of Guangdong General Hospital (the approval number: No. GDREC2010093A).

According to the methods described previously, we established a rat model of pressure-overload hypertrophy induced by abdominal aortic constriction (AAC) 20, a mouse model of pressure-overload hypertrophy induced by transverse aortic constriction (TAC) 21 and a mouse model of hypertrophy by subcutaneous injection of phenylephrine (PE) 22. Briefly, rats were anaesthetized with pentobarbital sodium at a dose of 35 mg/kg bodyweight intraperitoneally. The adequacy of anaesthesia was confirmed by the absence of reflex response to foot squeeze. Body temperature was maintained during surgery at 37 ± 0.5°C. At the end of the experiments, rats were killed with an overdose of sodium pentobarbital (220 mg/kg) intraperitoneal injection and hearts were collected for further detections. Consistently, rats were given the same anaesthesia and euthanasia as above in an *in vivo* study of overexpression of miR-16. Similarly, mice were anaesthetized intraperitoneally using sodium pentobarbital (50 mg/kg) before the TAC surgery, and mice were killed with an overdose of sodium pentobarbital (200 mg/kg) intraperitoneal injection.

### FITC-phalloidin staining and EU-Apollo staining

Cultured rat cardiomyocytes were washed in PBS, fixed for 10 min. in 3.7% formaldehyde, and permeabilized for 10 min. in 0.1% Triton X-100. Monolayers were then washed in blocking solution and incubated for 40 min. with FITC-phalloidin (10 μg/ml, Sigma-Aldrich) at 37°C. Monolayers were then washed again, post-fixed with 3.7% formaldehyde, and mounted. Based on the biosynthetic incorporation of the uridine analogue 5-ethynyluridine (EU) into newly transcribed RNA, the cellular transcription of NRVCs was analysed using a cell-light™ EU detection kit (RiboBio) according to the manufacturer's instructions. Confocal micrographs were obtained using a Leica SP5 confocal microscopy (Leica, Mannheim, Germany). Cell size (total area) was quantified using MiVnt imaging software (Weiyu, Zhuhai, China), and fluorescence intensity analysis was performed with the LAS AF-TCS SP5 imaging software.

### Culture of primary cardiomyocytes and treatment

Neonatal rat ventricular cells and neonatal mouse ventricular cells (NMVCs) were separately isolated from the hearts of 1–3-day-old newborn SD rats and C57BL6 mice as described previously 23. The newborn rats and mice were killed by cervical dislocation. Isolated NRVCs or NMVCs were plated onto 12-well plates and maintained for 48 hrs in DMEM/F-12 supplemented with 10% FBS (Gibco, New York, NY, USA). NRVCs were incubated with 2 × 10^−5^ M PE for 24 hrs and NMVCs were incubated with 10^−8^ M angiontensin II (Ang-II) for 48 hrs to induce the hypertrophic phenotype, respectively. As indicated, NRVCs were transduced with Adeno-empty or Adeno-miR-16 adenovirus (MOI 4). Total RNA and protein were extracted at 24 hrs after transduction.

### Quantitative mRNA and miRNA measurements

Quantitative real-time PCR (qRT-PCR) was performed as previously described 24. Methods for mRNA expression detection were as follows: Briefly, first-strand cDNAs were synthesized using a mixture of oligo (dT)_15_ and random primers with superscript reverse transcriptase (Invitrogen, Carlsbad, CA, USA). The absorption values of the SYBR Green I fluorescence in each tube were detected at the end of each thermal cycle. The housekeeping gene GAPDH was used as an internal control. Mature miR-16 level was detected using Bulge-Loop miRNA qRT-PCR kit (Ribobio). To normalize RNA content, the U6 snRNA was used as the internal control. PCR primers used in this study, as well as the size of fragments amplified, are shown in Table S2. Analyses were performed with a vii A7 Quantitative PCR System (Applied Biosystems, Carlsbad, CA, USA). Each sample was amplified in duplicate, and normalized *versus* the endogenous control. Results were calculated using the 2^−ΔΔCt^ method 25.

### Protein analysis

Protein extracts were obtained from cells or tissues, as described 24. The protein extract (40 μg) prepared from mouse myocardium was separated using 12% SDS-PAGE, transferred onto a polyvinylidene fluoride membrane, and probed with antibodies for ANP (1:500, cat. no. ab189921; Abcam (Cambridge, UK)), β-MHC (1:1000, cat. no. ab50967; Abcam), CCND1 (1:1000, cat. no. sc-246; Santa Cruz), CCND2 (1:1000, cat. no. sc-593; Santa Cruz (Santa Cruz, CA, USA)), CCNE1 (1:1000, cat. no. sc-481; Santa Cruz), p-pRb (1:1000, cat. no. sc-16671; Santa Cruz), pRb (1:1000, cat. no. sc-50; Santa Cruz), Stat3 (1:1000, cat. no. ab119352; Abcam), p-Stat3 (1:1000, cat. no. ab76315; Abcam), c-Myc (1:1000, cat. no. ab32072; Abcam), p-c-Myc (1:1000, cat. no. ab51156; Abcam) overnight at 4°C. Membranes were then washed extensively with TBS/T and incubated with a horseradish peroxidase-conjugated secondary antibody (1:5000, cat. no. sc-2370, sc-2371; Santa Cruz) for 1 hr at room temperature. Proteins were visualized using the ECL Plus detection system (GE Healthcare, Waukesha, WI, USA). As an internal control, membranes were also immunoblotted with an anti-GAPDH antibody (1: 2000, cat. no. sc-367715; Santa Cruz).

### Dual luciferase assay for CCND1, CCND2 and CCNE1 targets identification

As our previous report 26, the double-stranded DNA fragments containing the potential miR-16 binding site sequences of CCND1, CCND2 and CCNE1 genes were prepared. Then, the DNA fragments were introduced into the pGl3-promoter vector (Promega, Madison, WI, USA) to construct pGl3-target gene (TG)-binding site(bs) plasmid, respectively. Using a site-directed mutagenesis kit (TransGen, Beijing, China), the GCUGCU in the binding site for miR-16 in pGl3-TG-bs was replaced with GAAAAU to construct pGl3-TG-bs-MUT. The constructions of recombinant plasmids were identified by DNA sequencing.

Human embryonic kidney 293 cells (3 × 10^5^ cells per well) were plated in 12-well plates 24 hrs before transfection of plasmid DNA. Cells were cotransfected with 200 ng of pGl3-TG-bs or pGl3-TG-bs-MUT, 50 nM mir-16 mimic, and 20 ng of pRL-TK as an internal control (Promega). Activities of firefly luciferase (FL) and renal luciferase (RL) were measured 24 hrs after transfection, and the relative ratio of the FL/RL was used to determine miR-16-mediated knockdown of target genes.

### Statistical analysis

Results are expressed as means ± SD. In each experiment, all determinations were performed at least in triplicate. Differences between experimental groups were analysed with the unpaired Student's *t*-test. *P*-values lower than 0.05 were accepted as statistically significant.

## Results

### Down-regulation of miR-16 in hypertrophic myocardium

**Fig 1:**
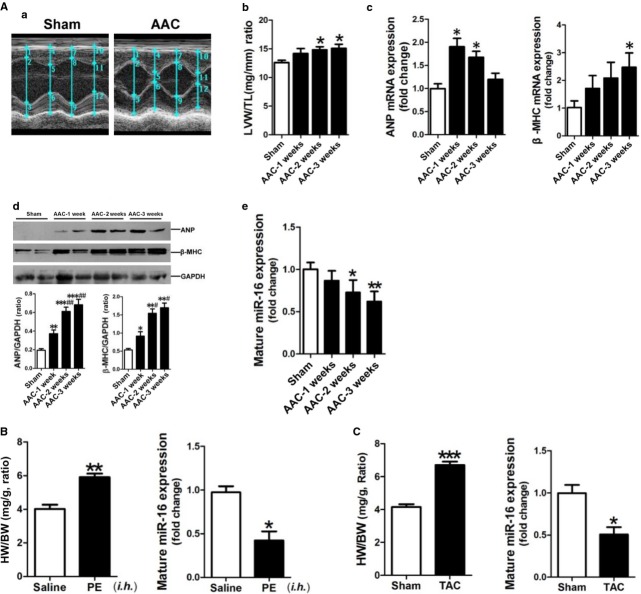
miR-16 is down-regulated in hypertrophic myocardium. (A) Establishment of a rat model of AAC-induced hypertrophy. (a) Representative echocardiographs showing that the LVAWd, LVAWs, LVPWd and LVPWs were increased in the hearts of rats in the AAC group. The detailed data were presented in Table S1. (b) The ratio of LV weight to tibial length. (c) ANP and β-MHC mRNA expressions were assessed by qRT-PCR assay (*n* + 6–8). (d) ANP and β-MHC protein expressions were assessed by Western blotting assay (*n* + 6–8). (e) Mature miR-16 expression was determined by qRT-PCR assay (*n* + 6–8). (B) The ratio of heart weight to bodyweight, and mature miR-16 expression in the myocardium of a mouse model of PE-induced cardiac hypertrophy by qRT-PCR (*n* + 6–8). The C57BL/6 mice were given subcutaneous injections of PE at 20 mg/kg/day for 3 days. The mice were then maintained for another 7 days, followed by cervical dislocation and removal of the hearts for subsequent analyses. (C) The ratio of heart weight to bodyweight, and mature miR-16 expression in the myocardium of a mouse model of TAC-induced cardiac hypertrophy as detected by qRT-PCR (*n* + 6–8). Data are shown as mean ± SD; *P < 0.05, **P < 0.01, ***P < 0.001 *versus* the Sham control or the Saline control, ^#^*P* < 0.05, ^##^*P* < 0.01 *versus* the AAC-1 w group, respectively.

Pressure-overload hypertrophy was induced by AAC in rats using 0.3-mm silver clips, as previously described 20. Echocardiography was performed to reveal cardiac structural changes in rats with and without AAC procedure (Fig.1A–a). The comparisons of all echocardiography findings were listed in Table S1. As expected, the systolic LV anterior wall thickness (LVAWs), the LV posterior wall end-diastolic thickness (LVPWd) and the LV posterior wall end-systolic thickness (LVPWs) were significantly increased in rats with AAC for 3 w compared with rats in the sham control group. However, the ejection fraction % and fractional shortening % were not yet significantly changed among the groups with AAC-1 w, -2 w and -3 w and the sham control group. Cardiac hypertrophy was further confirmed by the ratio of LV weight to tibial length (Fig.1A–b), the morphology (Fig. S1A-a) and the transverse slices (Fig. S1A-b) of the hearts of rats in the AAC-3 w group.

The results of qRT-PCR demonstrated that ANP mRNA was significantly up-regulated in myocardium of rats in the AAC-1 w and AAC-2 w groups, and β-MHC mRNA was significantly up-regulated in myocardium of rats in the AAC-3 w group (Fig.1A–c). Consistently, ANP and β-MHC protein expressions were markedly increased in myocardium of rats at as early as 1 week after AAC surgery, and were up-regulated more highly at 2 and 3 weeks after AAC surgery, respectively (Fig.1A–d). We next measured miR-16 expression in rat hypertrophic myocardium using qRT-PCR. Our results revealed that miR-16 was significantly decreased in myocardium of rats in the AAC-2 w and AAC-3 w groups compared with the sham group (Fig.1A–e). Consistently, results of *in situ* hybridization revealed that miR-16 level was significantly reduced in the myocardium of rats in the AAC-3 w group compared with the sham control group (Fig. S1A-c).

To further validate down-regulation of miR-16 in hypertrophic myocardium, we utilized two other mouse models of cardiac hypertrophy through subcutaneous injection of PE (Fig. S1B) and TAC surgery (Fig. S1C). As expected, significant down-regulation of miR-16 was also observed in hypertrophic myocardium in mice in both two mouse models (Fig.1B and C).

### Down-regulation of miR-16 in hypertrophic cardiomyocytes

**Fig 2:**
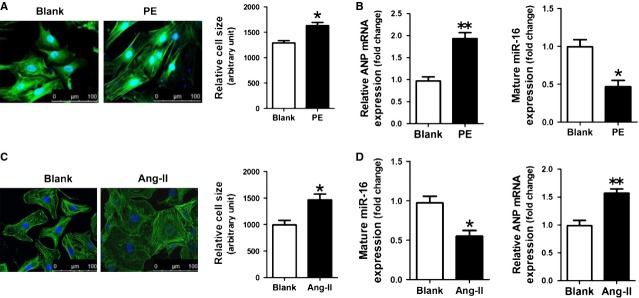
miR-16 is down-regulated in hypertrophic cardiomyocytes. (A) Morphologies of PE-treated NRVCs as revealed by FITC-phalloidin staining. (B) ANP mRNA and miR-16 expressions were assessed by qRT-PCR assay (*n* + 3). (C) Morphologies of Ang-II-treated NMVCs as revealed by FITC-phalloidin staining. (D) ANP mRNA and miR-16 expressions were assessed by qRT-PCR assay (*n* + 3). Data are shown as mean ± SD; *P < 0.05, **P < 0.01 *versus* the Blank control.

To investigate miR-16 expression in hypertrophic cardiomyocytes, we established a NRVC model of 20 mM PE-induced hypertrophy and a NMVC model of 10 nM Ang-II-induced hypertrophy. The FITC-phalloidin staining revealed a hypertrophic phenotype of NRVC and NMVC (Fig.2A and C). We measured ANP mRNA and miR-16 expression in the hypertrophic cardiomyocytes using qRT-PCR. Our results revealed that ANP mRNA was highly up-regulated, and miR-16 level was significantly reduced in the hypertrophic NRVCs and NMVCs compared with their control cells, respectively (Fig.2B and D).

### miR-16 inhibits cardiac hypertrophic phenotype *in vivo* and *in vitro*

**Fig 3:**
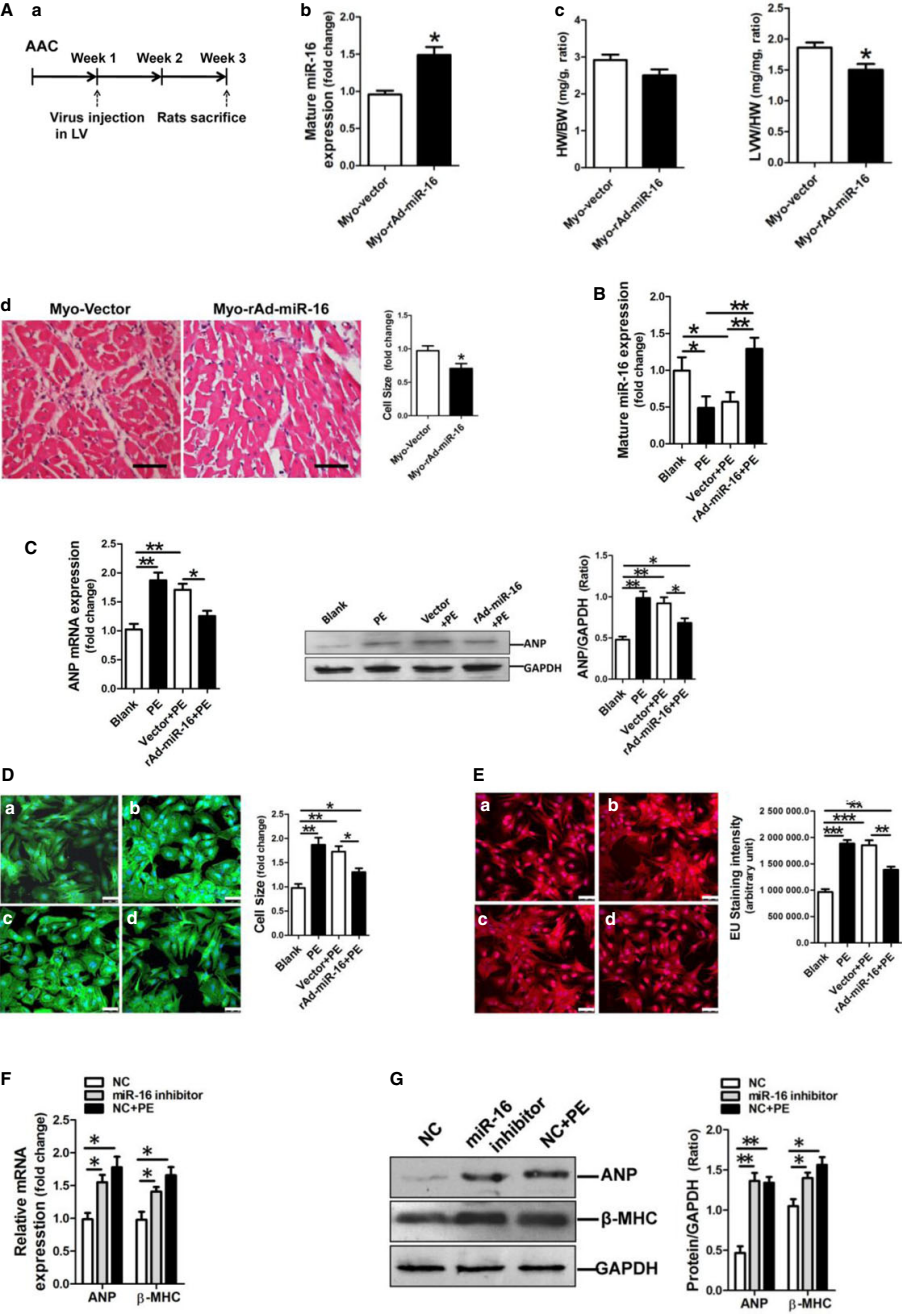
miR-16 inhibits cardiac hypertrophic phenotype *in vivo* and *in vitro*. (A) Adenovirus-mediated overexpression of miR-16 inhibited AAC-induced cardiac hypertrophy in rats (*n* + 6–8). (a) Schematic outline of AAC surgery and study time-points. (b) miR-16 expression in rat myocardium received virus injection was determined by qRT-PCR assay. (c) The ratios of heart weight to bodyweight and LV weight to heart weight. (d) Cell size of cardiomyocytes in rat myocardium received virus injection. miR-16 was overexpressed in the left ventricle, contributing to decreased LV mass. *P < 0.05 *versus* the vector control. (B) miR-16 expression in NRVCs was determined by qRT-PCR assay (*n* + 3). (C) ANP mRNA and protein expressions were assessed by assessed by qRT-PCR and Western blotting assay respectively. Restoration of miR-16 resulted in attenuation of PE-induced ANP up-regulation in NRVCs. (D) Cell size assessment of NRVCs by FITC-phalloidin staining assay. (a) Blank, (b) PE treatment, (c) Infection of the adenovirus vector control followed with PE incubation, (d) Infection of the adenovirus expressing miR-16 followed with PE incubation. The scale bar was 50 μm. (E) Assessment of newly transcribed RNA in NRVCs by EU-Apollo staining assay. NRVCs were treated differently in group (a), (b), (c) and (d), consistent with the corresponding groups in the above D. The scale bar was 50 μm. (F) ANP and β-MHC mRNA expressions in NRVCs were determined by qRT-PCR assay (*n* + 3). miR-16 inhibitor could increase ANP and β-MHC mRNA expressions in NRVCs. NC indicated negative control inhibitor. (G) ANP and β-MHC protein expressions were assessed by Western blotting assay. ANP protein expression was shown increased in miR-16 inhibitor-modified NRVCs. NC indicated the negative control inhibitor. Data are shown as mean ± SD; *P < 0.05, **P < 0.01, ***P < 0.001.

After establishing that miR-16 was down-regulated in hypertrophic myocardium, we investigated the effect of restoring miR-16 expression on the cardiac hypertrophic phenotype *in vivo*. To overexpress miR-16, we first prepared recombinant miR-16 adenovirus and control adenovirus (Fig. S2). Viral solution (5 × 10^7^ each) was injected into the LV myocardium of rats in AAC-1 w group at five sites respectively. After 2 weeks, rats with and without miR-16 overexpression were killed for further investigations (Fig.3A–a). As expected, high miR-16 level was confirmed in rat myocardium after 2-week infection with recombinant miR-16 adenovirus, but not the control virus (Fig.3A–b). Our results showed that the ratio of the LV weight to heart weight and cell size was significantly reduced in the hypertrophic myocardium with forced expression of miR-16 (Fig.3A–c and A–d).

Next, we investigated the effect of forced expression of miR-16 on the PE-induced hypertrophic phenotype in NRVCs. Quantification of miR-16 showed that miR-16 expression was restored in NRVCs in response to infection with recombinant miR-16 adenovirus (Fig.3B). Importantly, viral expression of miR-16 inhibited ANP mRNA and protein expressions in NRVCs induced by PE treatment (Fig.3C). Cell sizes and the levels of newly transcribed RNA in NRVCs were increased in response to treatment with PE, which were significantly inhibited by miR-16 overexpression (Fig.3D and E). To further confirm the effects of miR-16 on the hypertrophic phenotype in NRVCs, we transfected cells with 100 nM synthesized miR-16 inhibitor using oligofectamine reagent (Invitrogen). Interestingly, in contrast to the positive control with PE treatment, blockage of miR-16 function resulted in significant increase in ANP and β-MHC expressions at both mRNA and protein levels (Fig.3F and G).

### miR-16 targets CCND1, CCND2 and CCNE1, contributing to its repressing effect on cardiac hypertrophy

**Fig 4:**
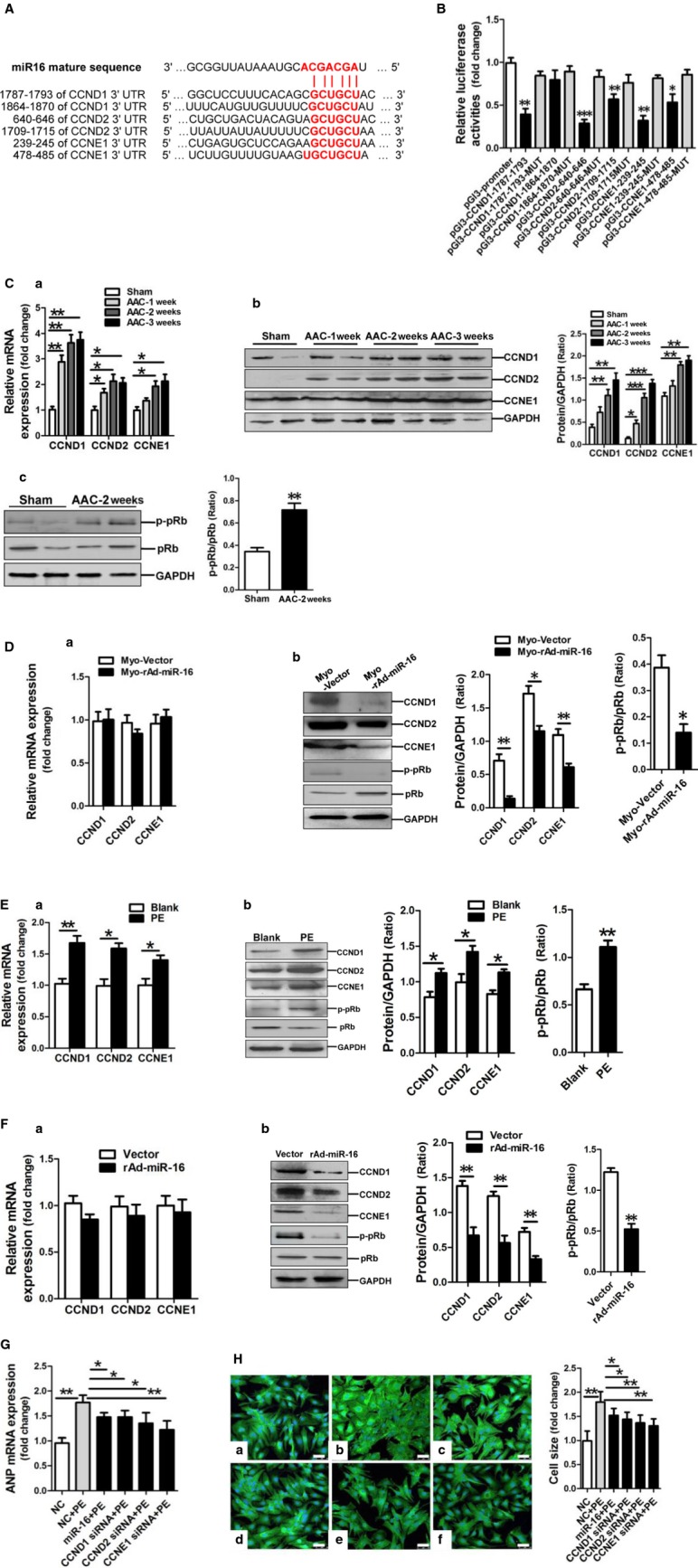
miR-16 negatively modulates the expression of CCND1, CCND2 and CCNE1. (A) Predicted miR-16 seed matches to potential target gene mRNAs. Results of *in silico* analysis suggesting the presence of miR-16 target sites on the genes of CCND1, CCND2 and CCNE1. The seed sequence of miR-16 is AGCAGCA, and the complementary nucleotide sequences are shown in bold words. (B) Verification of CCND1, CCND2 and CCNE1 as targets of miR-16 by the dual luciferase reporter system. Data on luciferase reporter activities show the interaction between miR-16 and 3′UTRs of target genes. MUT, indicates the mutated miR-16 binding site sequence GAAAAU instead of GCUGCU. Data are shown as mean ± SD; *P < 0.05, **P < 0.01, ***P < 0.001 *versus* pGl3-promoter vector control (*n* + 3). (C, a) CCND1, CCND2 and CCNE1 mRNA expressions in rat myocardium of AAC model of cardiac hypertrophy were assessed by qRT-PCR assay (*n* + 6–8). Levels of CCND1, CCND2 and CCNE1 protein expressions (b) and phosphorylated pRb (c) in rat myocardium of AAC model of cardiac hypertrophy were assessed by Western blotting assay (*n* + 6–8). (D, a) CCND1, CCND2 and CCNE1 mRNA expressions in rat myocardium received virus injection were assessed by qRT-PCR assay (*n* + 6–8). (b) Levels of CCND1, CCND2 and CCNE1 protein expressions and phosphorylated pRb in rat myocardium received virus injection were assessed by Western blotting assay (*n* + 6–8). (E, a) CCND1, CCND2 and CCNE1 mRNA expressions in PE-treated NRVCs were assessed by qRT-PCR assay (*n* + 3). (b) CCND1, CCND2 and CCNE1 protein expressions and phosphorylated pRb in PE-treated NRVCs were assessed by Western blotting assay (*n* + 3). (F, a) CCND1, CCND2 and CCNE1 mRNA expressions in miR-16-overexpressing NRVCs were assessed by qRT-PCR assay (*n* + 3). (b) Levels of CCND1, CCND2 and CCNE1 protein and phosphorylated pRb in miR-16-overexpressing NRVCs were assessed by Western blot assay (*n* + 3). (G) ANP mRNA expression was assessed by qRT-PCR assay in NRVCs modified with miR-16 mimic, CCND1 siRNA, CCND2 siRNA, and CCNE1 siRNA respectively (*n* + 3). (H) Cell size was assessed by FITC-phalloidin staining assay in NRVCs modified with miR-16 mimic, CCND1 siRNA, CCND2 siRNA, and CCNE1 siRNA respectively (*n* + 3). The negative control mimic (NC) were transfected into NRVCs (a), meanwhile, NC (b), miR-16 mimic (c), CCND1 siRNA (d), CCND2 siRNA (e) and CCNE1 siRNA (f) were also transfected in NRVCs followed with PE incubation, respectively. The scale bar was 50 μm. Data are shown as mean ± SD; *P < 0.05, **P < 0.01, ***P < 0.001.

By analysing the databases Tarbase (www.diana.imis.athena-innovation.gr), Mirdb (www.mirdb.org) and TargetScan-Vert (www.targetscan.org), we found that the target sites for miR-16 have been observed on the following genes: CCND1, CCND2 and CCNE1. The matching positions for miR-16 within 3′UTR of the targeted mRNAs are shown in Figure4A. The dual luciferase assay demonstrated that miR-16 significantly reduced the luciferase activities through binding the following sites, including 1787–1793 of CCND1 3′UTR, 1864–1870 of CCND1 3′UTR, 640–646 of CCND2 3′UTR, 1709–1715 of CCND2 3′UTR, 239–245 of CCNE1 3′UTR and 478–485 of CCNE1 3′UTR (Fig.4B).

Next, we detected the expressions of CCND1, CCND2 and CCNE1 in myocardium of rats with AAC. As expected, our results of qRT-PCR and Western blotting assay revealed that the expressions of CCND1, CCND2 and CCNE1 were significantly up-regulated in myocardium of rats in AAC-2 w and AAC-3 w groups (Fig.4C–a and C–b). Consistently, the level of phosphorylated pRb was also increased in the hypertrophic myocardium (Fig.4C-c). In addition, we determined expressions of CCND1, CCND2, CCNE1 and pRb in rat hypertrophic myocardium after 2-week infection with recombinant miR-16 adenovirus. Compared with the control vector, adenovirus-mediated overexpression of miR-16 markedly decreased the levels of CCND1, CCND2, CCNE1 proteins and phosphorylated pRb, with no effect on mRNA expressions of CCND1, CCND2 and CCNE1(Fig.4D–a and D–b).

In parallel to a rat model of AAC-induced hypertrophy, expressions of CCND1, CCND2 and CCNE1 at both mRNA and protein levels were significantly up-regulated in PE-treated NRVCs, with increased level of phosphorylated pRb (Fig.4E–a and E–b) and increased cell population of S phase (Fig. S3A). We next determined whether the expressions of CCND1, CCND2 and CCNE1 genes were down-regulated by miR-16 overexpression. Hence, NRVCs were infected without (empty vector) and with recombinant miR-16-encoding adenovirus for 24 hrs. Our data showed that the expressions of CCND1, CCND2, CCNE1 proteins and phosphorylated pRb were significantly reduced (Fig.4F–b) and the cell population of S phase was also decreased (Fig. S3B) in NRVCs following overexpression of miR-16, but mRNA levels of CCND1, CCND2 and CCNE1 remained unchanged (Fig.4F–a). Collectively, miR-16 reduces the expression of CCND1, CCND2 and CCNE1 in NRVCs at the post-transcriptional level.

Furthermore, we transfected NRVCs with miR-16 mimic, siRNA for CCND1, CCND2 or CCNE1, respectively, followed by examining ANP mRNA expression and morphologies of NRVCs. Our results showed that ANP mRNA was down-regulated consistently in PE-treated NRVCs in response to transfection with miR-16 mimic, CCND1 siRNA, CCND2 siRNA and CCNE1 siRNA respectively (Fig.4G). FITC-phalloidin staining results demonstrated that cell size changes of PE-treated NRVCs were also significantly reversed after transfection with miR-16 mimic, or siRNA for CCND1, CCND2 or CCNE1, respectively (Fig.4H). These results suggest that either miR-16 overexpression or knockdown of CCND1, CCND2 or CCNE1 can similarly inhibit PE-induced hypertrophic phenotype of NRVCs. Taken together, these data indicate that miR-16 down-regulation resulted in an increase in CCND1, CCND2 and CCNE1, and activation of cyclin/Rb pathway in hypertrophic myocardium.

### miR-16 is down-regulated through the STAT3/c-Myc pathway

**Fig 5:**
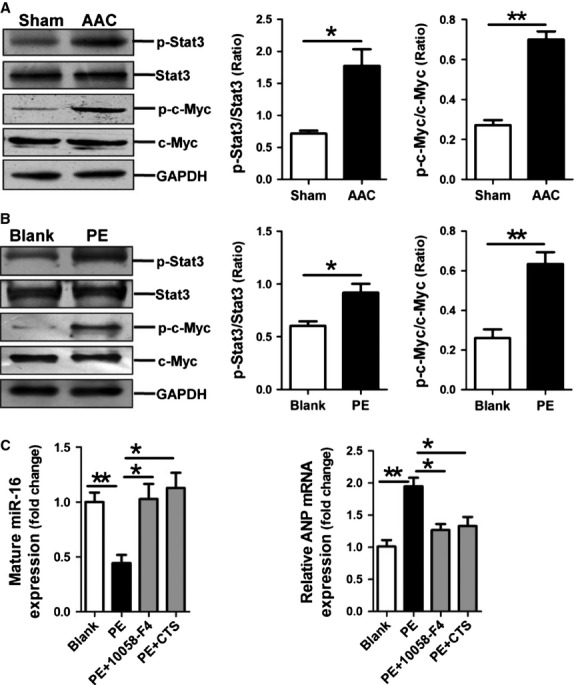
The STAT3/c-Myc pathway mediates miR-16 down-regulation during cardiac hypertrophy. (A) Activation of STAT3 and c-Myc in myocardial hypertrophy in rats receiving AAC treatment for 2 weeks (*n* + 5–7). (B) Activation of STAT3 and c-Myc in PE-treated NRVCs (*n* + 3). (C) miR-16 and ANP mRNA expressions were assessed by qRT-PCR assay in PE-treated NRVCs without and with the presence of STAT3 inhibitor cryptotanshinone (10 μM) and c-Myc inhibitor 10058-F4 (30 μM), respectively (*n* + 3). Data are shown as mean ± SD; *P < 0.05, **P < 0.01, ***P < 0.001.

Previous study revealed that STAT3/c-Myc pathway mediated progestin-induced suppression of miR-16 expression in mammary tumour cells 27. Therefore, we investigated whether miR-16 down-regulation in cardiac hypertrophy was also mediated through activation of the STAT3/c-Myc pathway. We first detected activation of STAT3/c-Myc pathway in both myocardium of rats with AAC and NRVCs treated with PE. Western blot results showed that phosphorylation levels of STAT3 and c-Myc were significantly increased in myocardium of rats with AAC and in PE-treated NRVCs compared with their controls, respectively (Fig.5A and B), suggesting activation of the STAT3/c-Myc pathway during cardiac hypertrophy. Next, we pre-treated NRVCs in the presence of PE with 10058-F4 (c-Myc inhibitor) or with cryptotanshinone (STAT3 inhibitor) for 0.5 hr, followed by analysis with qRT-PCR assay. Our results demonstrated that treatment with either 10058-F4 or cryptotanshinone prevented the down-regulation of miR-16 induced by treatment with PE (Fig.5C). Consistently, the up-regulation of ANP mRNA expression in PE-treated NRVCs was also inhibited by treatment with 10058-F4 or cryptotanshinone (Fig.5C). Collectively, miR-16 down-regulation in hypertrophic cardiomyocytes results from activation of the STAT3/c-Myc pathway.

## Discussion

In this study, we have, for the first time, shown that miR-16 was down-regulated in hypertrophic cardiomyocytes *in vivo* and *in vitro*, which was accompanied by up-regulation of CCND1, CCND2, CCNE1 and activation of cyclin/Rb pathway. Overexpression of miR-16 down-regulated CCND1, CCND2 and CCNE1 at the post-transcriptional level. We have provided further evidence to support that miR-16 inhibited cardiac hypertrophy through targeting CCND1, CCND2, CCNE1 and cyclin/Rb pathway. Our results have also suggested a role of the STAT3/c-Myc pathway in miR-16 down-regulation during cardiac hypertrophy.

It is reported that CCND-CDK4/6 phosphorylated pRb during hypertrophy and expression of an unphosphorylatable pRb mutant could impair hypertrophic growth in cardiomyocytes 9. CCND1-Rb and CCND2-Rb pathways were demonstrated to be crucial in cardiomyocyte hypertrophy 7,28. pRb and the E2F family of transcription factors were also shown to play important roles in the development of cardiac hypertrophy 7,9. Once inactivated by phosphorylation, pRb will no longer repress the activation of E2F which is necessary for hypertrophic growth in cardiomyocytes 9,29.

More specifically, several lines of evidence derived from our studies support that miR-16 inhibits cardiac hypertrophy through targeting CCND1, CCND2 and CCNE1. First, miR-16 is highly expressed in cardiomyocytes 30, and negatively regulates cellular growth and cell cycle progression 31. The role of miR-16 was also previously implicated in cell cycle regulation of cancer cells 32, neural differentiation 33 and mesenchymal stem cell differentiation towards myogenic phenotype 34. In this study, we have shown that down-regulation of miR-16 was accompanied by up-regulations of CCND1, CCND2 and CCNE1 in hypertrophic myocardium and cell models of cardiac hypertrophy. Importantly, knockdown of endogenous miR-16 in cardiomyocytes increased the expression of ANP and β-MHC. Second, enforced expression of miR-16 through adenovirus inhibited cardiac growth in rat AAC model. Overexpression of miR-16 or siRNA knockdown of CCND1, CCND2 or CCNE1 inhibited ANP up-regulation and size increases of cardiomyocytes. All these results have further indicated that miR-16 post-transcriptionally modulates CCND1, CCND2 and CCNE1 expressions, which is consistent with previous reports 32,34,35. Given that growth of cardiac myocytes results from activation of various signalling pathways and increases in gene transcription and protein synthesis 36,37, our results showing that overexpression of miR-16 reduced the newly transcribed RNA in PE-treated NRVCs further support that miR-16 inhibits cardiac hypertrophy.

In addition, the roles of cyclins and CDKs have been previously proposed in cardiac hypertrophy. For example, cyclin D expression and CDK activity were induced in hypertrophic cardiomyocytes *in vitro* 6–8,10,38 and *in vivo* 6,8,10. And inhibiting cyclin/CDK activity in cardiac myocytes can attenuate hypertrophic growth 38,39.

Consistently, this study showed that CCND1 and CCND2 were up-regulated at both mRNA and protein levels in rat myocardium of AAC model and PE-stimulated NRVCs. It was previously reported that cyclin E was expressed in left ventricles of newborn animals, but undetectable in adult ventricles 8. However, our results demonstrated that CCNE1 was highly expressed in both NRVCs and adult rat ventricles, and was also up-regulated at both mRNA and protein levels in rat hypertrophic myocardium and in PE-stimulated NRVCs, suggesting a novel role for CCNE1 in the development of hypertrophy. Therefore, this study demonstrates that increased expression of CCND1, CCND2 and CCNE1 were modulated at both transcriptional level and post-transcriptional level during cardiac hypertrophy.

Notably, our findings showing that down-regulation of miR-16 results in cardiac hypertrophy through up-regulating the expressions of CCND1, CCND2 and CCNE1 have raised many other questions. For example, what triggers down-regulation of miR-16 during hypertrophy? We have attempted to address this question. Our results have suggested a role of the STAT3/c-Myc pathway in miR-16 down-regulation during hypertrophy, as pharmacological inhibition of either STAT3 or c-Myc prevented the down-regulation of miR-16 and subsequent hypertrophy. This conclusion has been supported by previous studies showing that STAT3 activation is involved in the development of cardiac hypertrophy 40–43. In addition, a previous study reported that the STAT3/c-Myc pathway mediated miR-16 suppression by progestin in mammary tumour cells 27, further supporting our conclusion.

**Fig 6:**
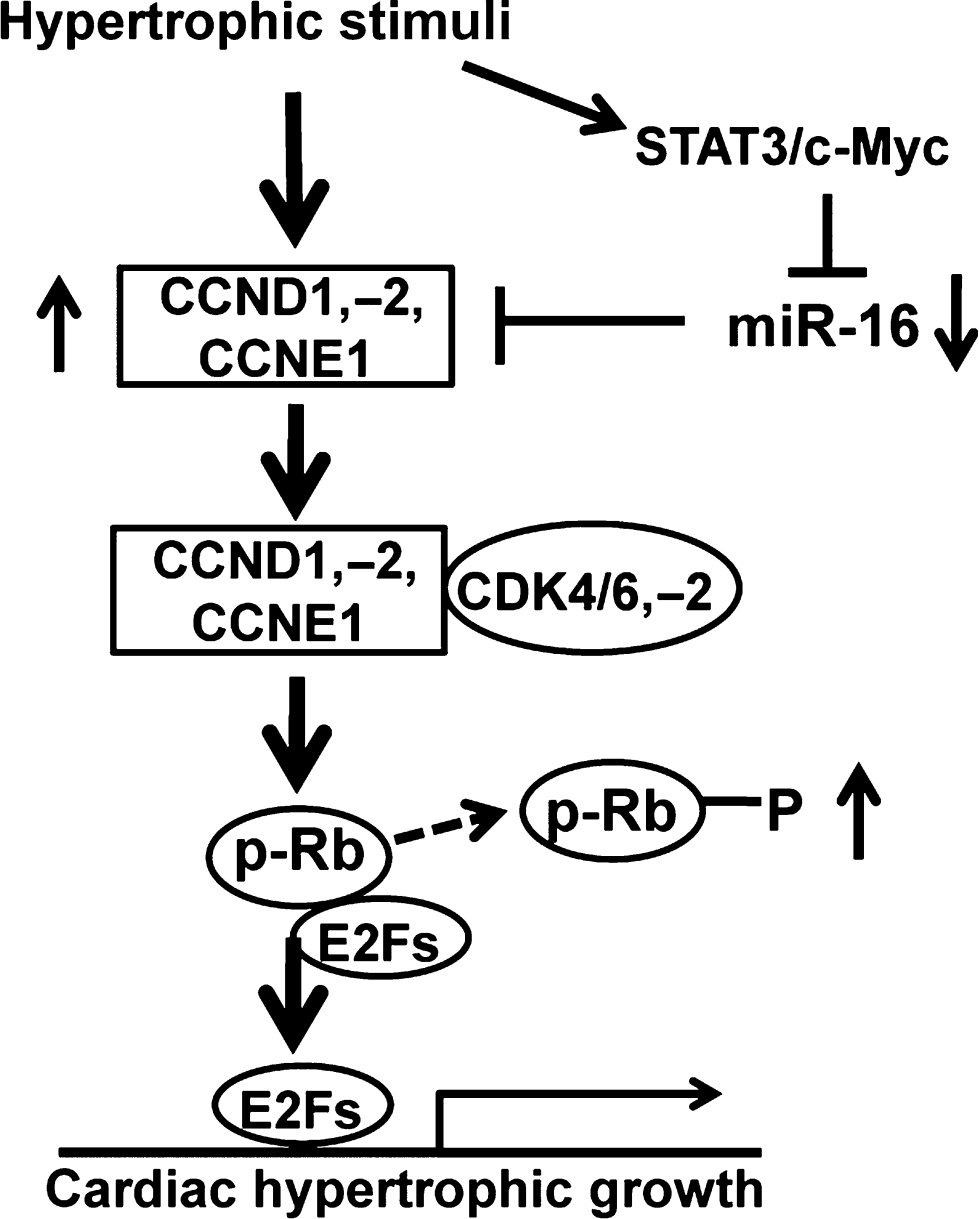
Schematic diagram of the mechanism whereby miR-16 attenuation contributes to myocardial hypertrophy. Activation of the STAT3/c-Myc signalling pathway negatively regulates miR-16 expression during cardiac hypertrophy. In addition, attenuation of miR-16 derepresses the cyclins D1, D2 and E1 and activates cyclin/Rb pathway to provoke cardiomyocyte hypertrophy.

Taken together, our results have revealed that miR-16 was down-regulated in cardiomyocytes during cardiac hypertrophy, and overexpression of miR-16 could ameliorate hypertrophic phenotype in cardiomyocytes. Our data have also revealed that miR-16 inhibits hypertrophic phenotype in cardiomyocytes through down-regulation of CCND1, CCND2 and CCNE1 expression and inactivation of cyclin/Rb pathway (Fig.6). Therefore, we conclude that miR-16 might be a potential target for prevention and treatment of cardiac hypertrophy.

## Supporting Information

**Figure S1** Animal models of cardiac hypertrophy.

**Figure S2** Preparation of recombinant miR-16 adenovirus.

**Figure S3** Cell cycle distribution of NRVCs.

**Table S1** Assessment of the cardiac function by echocardiography.

**Table S2** Primers used in qRT-PCR assay.

## References

[b1] Chien KR, Grace AA, Hunter JJ, Chien KR (1999). Molecular and cellular biology of cardiac hypertrophy and failure. Molecular basis of cardiovascular disease.

[b2] Perrino C, Naga Prasad SV, Mao L (2006). Intermittent pressure overload triggers hypertrophy-independent cardiac dysfunction and vascular rarefaction. J Clin Invest.

[b3] van Berlo JH, Maillet M, Molkentin JD (2013). Signaling effectors underlying pathologic growth and remodeling of the heart. J Clin Invest.

[b4] Sadoshima J, Aoki H, Izumo S (1997). Angiotensin II and serum differentially regulate expression of cyclins, activity of cyclin-dependent kinases, and phosphorylation of retinoblastoma gene product in neonatal cardiac myocytes. Circ Res.

[b5] Poolman RA, Brooks G (1998). Expressions and activities of cell cycle regulatory molecules during the transition from myocyte hyperplasia to hypertrophy. J Mol Cell Cardiol.

[b6] Li JM, Poolman RA, Brooks G (1998). Role of G1 phase cyclins and cyclin-dependent kinases during cardiomyocyte hypertrophic growth in rats. Am J Physiol.

[b7] Nozato T, Ito H, Tamamori M (2000). G1 cyclins are involved in the mechanism of cardiac myocyte hypertrophy induced by angiotensin II. Jpn Circ J.

[b8] Busk PK, Bartkova J, Strøm CC (2002). Involvement of cyclin D activity in left ventricle hypertrophy *in vivo* and *in vitro*. Cardiovasc Res.

[b9] Hinrichsen R, Hansen AH, Haunsø S (2008). Phosphorylation of pRb by cyclin D kinase is necessary for development of cardiac hypertrophy. Cell Prolif.

[b10] Hotchkiss A, Robinson J, MacLean J (2012). Role of D-type cyclins in heart development and disease. Can J Physiol Pharmacol.

[b11] Bartel DP (2004). MicroRNAs: genomics, biogenesis, mechanism, and function. Cell.

[b12] Carè A, Catalucci D, Felicetti F (2007). MicroRNA-133 controls cardiac hypertrophy. Nat Med.

[b13] Callis TE, Pandya K, Seok HY (2009). MicroRNA-208a is a regulator of cardiac hypertrophy and conduction in mice. J Clin Invest.

[b14] Li Q, Song XW, Zou J (2010). Attenuation of microRNA-1 derepresses the cytoskeleton regulatory protein twinfilin-1 to provoke cardiac hypertrophy. J Cell Sci.

[b15] Da Costa Martins PA, De Windt LJ (2012). MicroRNAs in control of cardiac hypertrophy. Cardiovasc Res.

[b16] Ucar A, Gupta SK, Fiedler J (2012). The miRNA-212/132 family regulates both cardiac hypertrophy and cardiomyocyte autophagy. Nat Commun.

[b17] Ganesan J, Ramanujam D, Sassi Y (2013). MiR-378 controls cardiac hypertrophy by combined repression of mitogen-activated protein kinase pathway factors. Circulation.

[b18] Heymans S, Corsten MF, Verhesen W (2013). Macrophage microRNA-155 promotes cardiac hypertrophy and failure. Circulation.

[b19] Huang ZP, Chen J, Seok HY (2013). MicroRNA-22 regulates cardiac hypertrophy and remodeling in response to stress. Circ Res.

[b20] Phrommintikul A, Tran L, Kompa A (2008). Effects of a Rho kinase inhibitor on pressure overload induced cardiac hypertrophy and associated diastolic dysfunction. Am J Physiol Heart Circ Physiol.

[b21] Rockman HA, Ono S, Ross RS (1994). Molecular and physiological alterations in murine ventricular dysfunction. Proc Natl Acad Sci USA.

[b22] Slawson SE, Roman BB, Williams DS (1998). Cardiac MRI of the normal and hypertrophied mouse heart. Magn Reson Med.

[b23] Communal C, Singh K, Pimentel D (1998). Norepinephrine stimulates apoptosis in adult rat ventricular myocytes by activation of the beta-adrenergic pathway. Circulation.

[b24] Zhu JN, Chen R, Fu YH (2013). Smad3 inactivation and MiR-29b upregulation mediate the effect of carvedilol on attenuating the acute myocardium infarction-induced myocardial fibrosis in rat. PLoS ONE.

[b25] Pfaffl MW (2001). A new mathematical model for relative quantification in real-time RT-PCR. Nucleic Acids Res.

[b26] Shan ZX, Lin QX, Deng CY (2010). miR-1/miR-206 regulate Hsp60 expression contributing to glucose-mediated apoptosis in cardiomyocytes. FEBS Lett.

[b27] Rivas MA, Venturutti L, Huang YW (2012). Downregulation of the tumor-suppressor miR-16 *via* progestin-mediated oncogenic signaling contributes to breast cancer development. Breast Cancer Res.

[b28] Busk PK, Hinrichsen R (2003). Cyclin D in left ventricle hypertrophy. Cell Cycle.

[b29] Vara D, Bicknell KA, Coxon CH (2003). Inhibition of E2F abrogates the development of cardiac myocyte hypertrophy. J Biol Chem.

[b30] Duisters RF, Tijsen AJ, Schroen B (2009). miR-133 and miR-30 regulate connective tissue growth factor: implications for a role of microRNAs in myocardial matrix remodeling. Circ Res.

[b31] Linsley PS, Schelter J, Burchard J (2007). Transcripts targeted by the microRNA-16 family cooperatively regulate cell cycleprogression. Mol Cell Biol.

[b32] Liu Q, Fu H, Sun F (2008). miR-16 family induces cell cycle arrest by regulating multiple cell cycle genes. Nucleic Acids Res.

[b33] Aranha MM, Santos DM, Xavier JM (2010). Apoptosis-associated microRNAs are modulated in mouse, rat and human neural differentiation. BMC Genomics.

[b34] Liu JL, Jiang L, Lin QX (2012). MicroRNA 16 enhances differentiation of human bone marrow mesenchymal stem cells in a cardiac niche toward myogenic phenotypes *in vitro*. Life Sci.

[b35] Cui X, Witalison EE, Chumanevich AP (2013). The induction of microRNA-16 in colon cancer cells by protein arginine deiminase inhibition causes a p53-dependent cell cycle arrest. PLoS ONE.

[b36] Hannan RD, Stefanovsky V, Taylor L (1996). Overexpression of the transcription factor UBF1 is sufficient to increase ribosomal DNA transcription in neonatal cardiomyocytes: implications for cardiac hypertrophy. Proc Natl Acad Sci USA.

[b37] Bernardo BC, Weeks KL, Pretorius L (2010). Molecular distinction between physiological and pathological cardiac hypertrophy: experimental findings and therapeutic strategies. Pharmacol Ther.

[b38] Tamamori M, Ito H, Hiroe M (1998). Essential roles for G1 cyclin-dependent kinase activity in development of cardiomyocyte hypertrophy. Am J Physiol.

[b39] Nozato T, Ito H, Watanabe M (2001). Overexpression of cdk inhibitor p16INK4a by adenovirus vector inhibits cardiac hypertrophy *in vitro* and *in vivo*: a novel strategy for the gene therapy of cardiac hypertrophy. J Mol Cell Cardiol.

[b40] Frias MA, Rebsamen MC, Gerber-Wicht C (2008). Prostaglandin E2 activates Stat3 in neonatal rat ventricular cardiomyocytes: a role in cardiac hypertrophy. Cardiovasc Res.

[b41] Willey CD, Palanisamy AP, Johnston RK (2008). STAT3 activation in pressure-overloaded feline myocardium: role for integrins and the tyrosine kinase BMX. Int J Biol Sci.

[b42] Li Y, Zhang H, Liao W (2011). Transactivated EGFR mediates α1-AR-induced STAT3 activation and cardiac hypertrophy. Am J Physiol Heart Circ Physiol.

[b43] Leifheit-Nestler M, Wagner NM, Gogiraju R (2013). Importance of leptin signaling and signal transducer and activator of transcription-3activation in mediating the cardiac hypertrophy associated with obesity. J Transl Med.

