# Supplementary figures and images for: Attenuation of microRNA-16 derepresses the cyclins D1, D2 and E1 to provoke cardiomyocyte hypertrophy
Source: J Cell Mol Med. 2015 Jan 13;19(3):608–19. doi: 10.1111/jcmm.12445 (PMC4369817; doi:10.1111/jcmm.12445)

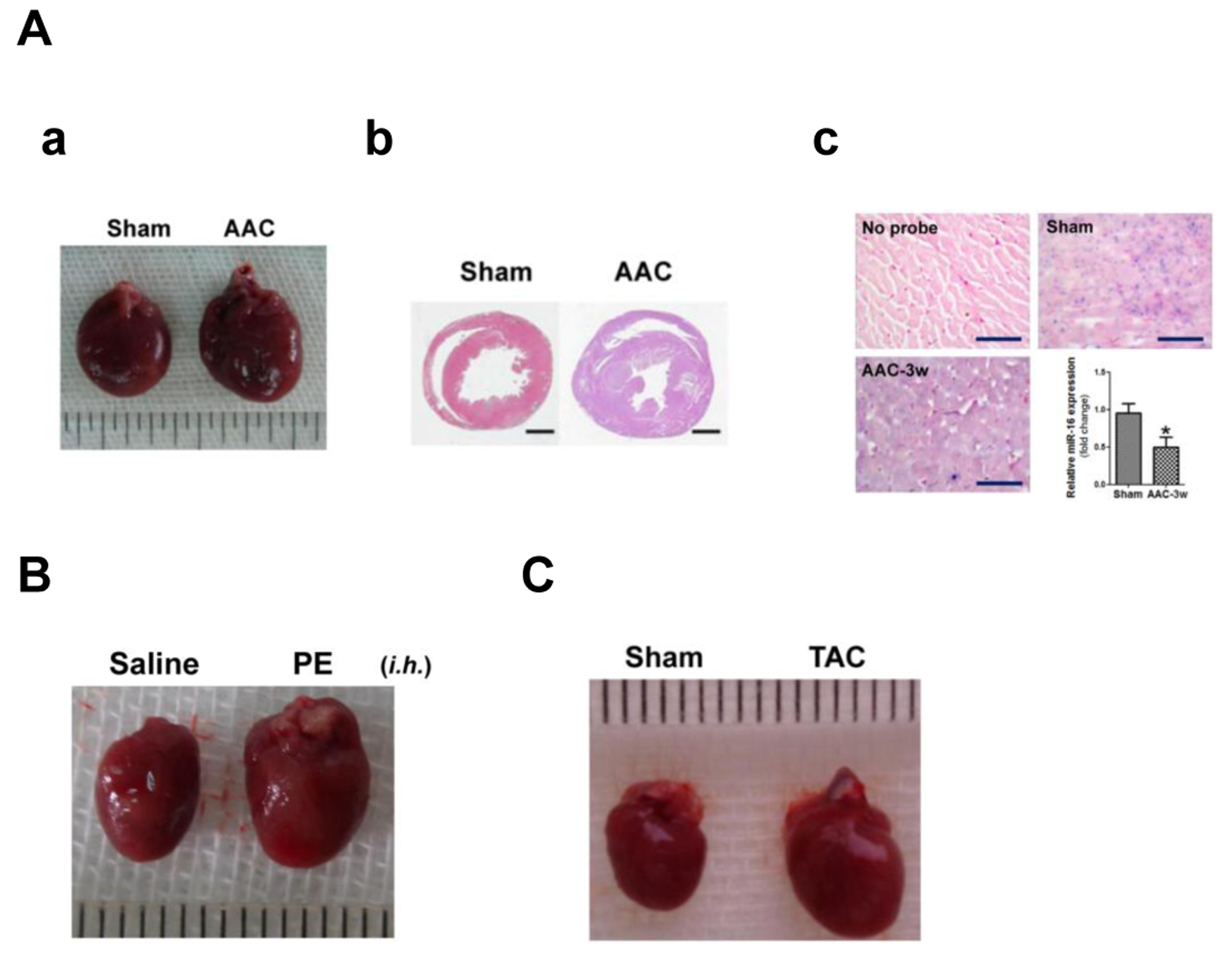

Supplement: Supplementary file 1 [file jcmm0019-0608-sd1.tif]

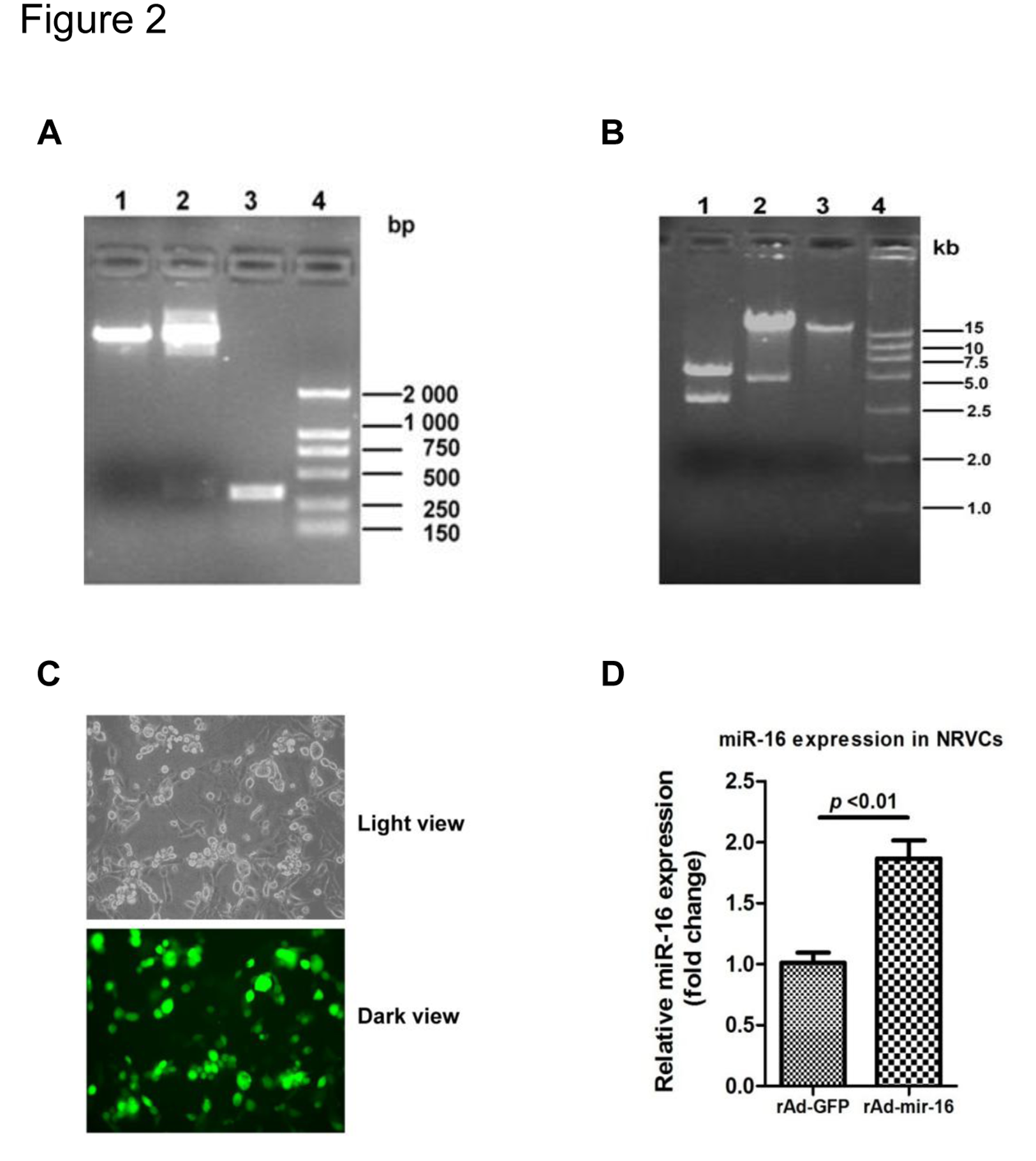

Supplement: Supplementary file 2 [file jcmm0019-0608-sd2.tif]

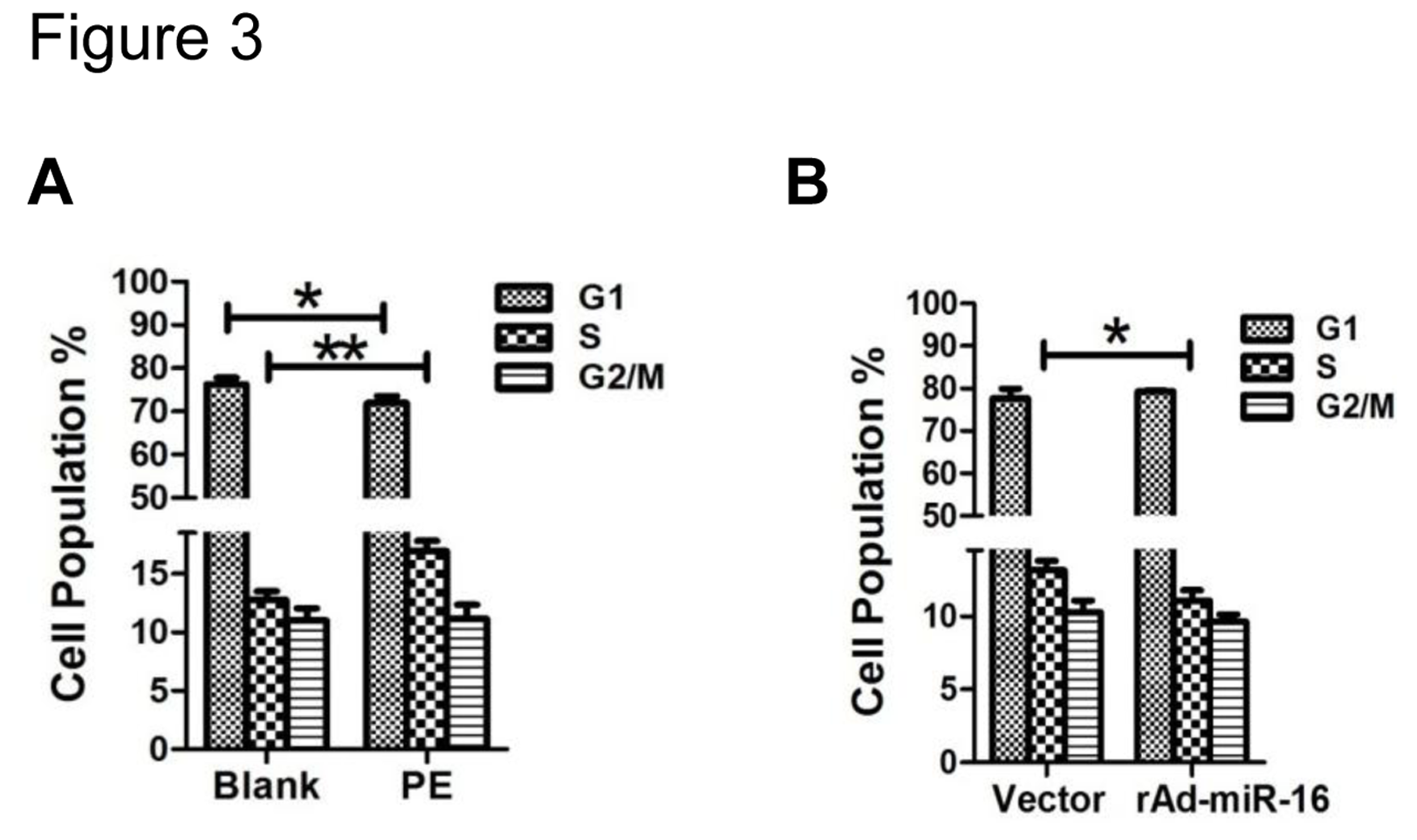

Supplement: Supplementary file 3 [file jcmm0019-0608-sd3.tif]
