# Supplementary material for: Attenuation of microRNA-16 derepresses the cyclins D1, D2 and E1 to provoke cardiomyocyte hypertrophy
Source: J Cell Mol Med. 2015 Jan 13;19(3):608–19. doi: 10.1111/jcmm.12445 (PMC4369817; doi:10.1111/jcmm.12445)
Supplement: Supplementary file 4 [file jcmm0019-0608-sd4.doc]

**Supplementary**

**Table 1.**

**Assessment of the cardiac function by echocardiography**

| Group | Sham | AAC-1w | AAC-2w | AAC-3w |
| --- | --- | --- | --- | --- |
| LVAWd | 1.65±0.18 | 1.54±0.20 | 1.77±0.12 | 1.87±0.21 |
| LVAWs | 2.20±0.29 | 2.63±0.35^*^ | 2.82±0.28^**^ | 2.87±0.29^**^ |
| LVIDd | 6.43±0.58 | 6.66±0.62 | 6.42±0.70 | 6.66±0.81 |
| LVIDs | 4.11±0.92 | 4.35±0.48 | 3.87±1.15 | 4.22±0.96 |
| LVPWd | 1.64±0.18 | 1.78±0.24 | 1.88±0.46 | 2.02±0.15^*^ |
| LVPWs | 2.53±0.31 | 2.54±0.15 | 2.80±0.34 | 3.01±0.48^*^ |
| EF (%) | 64.5±6.04 | 62.3±10.9 | 69.6±6.37 | 65.1±9.90 |
| FS (%) | 36.18±3.72 | 34.38±7.75 | 39.76±3.10 | 35.69±3.85 |

Data represent the mean±SD, ^*^*p* < 0.05, ^**^*p* < 0.01 versus sham group, N=6-8.
