# Supplementary material for: Attenuation of microRNA-16 derepresses the cyclins D1, D2 and E1 to provoke cardiomyocyte hypertrophy
Source: J Cell Mol Med. 2015 Jan 13;19(3):608–19. doi: 10.1111/jcmm.12445 (PMC4369817; doi:10.1111/jcmm.12445)
Supplement: Supplementary file 5 [file jcmm0019-0608-sd5.doc]

**Supplementary Table 2.**

**Primers used in qRT-PCR assay**

| Gene | Sequence (5′- 3′) | Product size (bp） |
| --- | --- | --- |
| *ANP* | F, CTGAGGTGCCTCCCTGGAC  R, AGTCCGCTCTGGGCTCCAA | 199 |
| *β-MHC* | F, CAAGGAGCTCACCTACCAGA  R, CAGCTTGTTGACCTGGGACT | 200 |
| *CCND1* | F, CCCCAACAACTTCCTCTCCT  R, CAAGCCAGACCAGCTTCTTC | 198 |
| *CCND2* | F, TCATCGCTCTGTGTGCTACC  R, CTGGCAGGCTTTGAGACAAT | 191 |
| *CCNE1* | F, CTCGTTGGAGTTGATGCAGA  R, CTTTCTTTGCTTGGGCTTTG | 209 |
| *GAPDH*  Mature  miR-16  U6 | F, CAAGAAGGTGGTGAAGCAGG  R, CCACCCTGTTGCTGTAGCC  RT, GTCGTATCCAGTGCGTGTCGTGGAGT  CGGCAATTGCACTGGATACGACCGCCAATA  F, GTCCGCTAGCAGCACGTAAATATT  R, GTGCGTGTCGTGGAGTC  RT, GTCGTATCCAGTGCGTGTCGTGGAGT  CGGCAATTGCACTGGATACGAC  F, GTCCGCGTGCTCGCTTCGGCAGC  R, GTGCGTGTCGTGGAGTC | 200  76  160 |
