# Supplementary material for: Attenuation of microRNA-16 derepresses the cyclins D1, D2 and E1 to provoke cardiomyocyte hypertrophy
Source: J Cell Mol Med. 2015 Jan 13;19(3):608–19. doi: 10.1111/jcmm.12445 (PMC4369817; doi:10.1111/jcmm.12445)
Supplement: Supplementary file 6 [file jcmm0019-0608-sd6.docx]

**Supporting information**

**1 Supplementary methods**

**Echocardiography assay**

Left ventricular (LV) structure and function variables were assessed by transthoracic echocardiography. After the induction of light general anesthesia, rats underwent transthoracic two dimensional (2D) guided M-mode echocardiography with a 8.5-MHz transducer (Acuson, Mountain View, CA). From the cardiac short axis (papillary level), the LV anterior wall end diastolic thickness, the systolic LV anterior wall thickness, the LV internal dimension at end-diastole, the LV internal dimension at end-systole, the LV posterior wall end-diastolic thickness, the LV posterior wall end-systolic thickness, the ejection fraction and fractional shortening were measured. Echocardiographic measurements were averaged from at least three separate cardiac cycles.

**MiR-16 *in situ* hybridization**

*In situ* hybridization for miR-16 was performed on paraffin sections of rat myocardium without or with a 5′-end biotin-labeled miR-16 detection probe. Briefly, 4-μm paraffin sections were dewaxed, hydrated and incubated with 40 μg/ml protein K at room temperature for 20 min. After washing with PBS containing 0.2% glycine for 3 times, the sections were fixed in 4% paraformaldehyde for 10 min then hybridized with 10 nmol/L miR-16 detection probes (Guangzhou JIGE, China) in hybridization buffer (60% formamide, 5XSSC, 9.2mM citric acid solution (pH6.0), 0.5 mg/mL yeast RNA, 50 μg/ml Heparin, 0.1%Tween-20) at 37°C overnight. After washing and blocking with 2% sheep serum, and 2 mg/ml BSA in PBST, the sections were incubated with an anti–biotin-AP (1:1000, Roche) for 1 h at room temperature. After washing in PBS, the sections were incubated with BCIP/NBT in dark overnight. The sections were viewed and images were captured under standard bright field microscopy.

**2 Supplementary legends**

**Table 1. Assessment of the cardiac function by echocardiography**

Data represent the mean±SD, **p* < 0.05, ***p* < 0.01 *vs* the sham group, *n*=6-8.

**Table 2. Primers used in qRT-PCR assay**

**Figure 1** Animal models of cardiac hypertrophy. (*A*) Establishment of a rat model of abdominal aortic constriction (AAC)-induced hypertrophy. (*a*) The morphologies (*a*) and the transverse slices (*b*) of rat hearts in the Sham group and the AAC-3 w group. The scale bar was 20 mm. (*c*) MiR-16 detection in rat myocardium by *in situ* hybridization assay. The scale bar was 50 μm. (*B*) The morphology of mouse heart of a mouse model of PE-induced cardiac hypertrophy. (*C*) The morphology of mouse heart of a mouse model of TAC-induced cardiac hypertrophy.

**Figure 2** Preparation of recombinant miR-16 adenovirus.

(*A*) Restriction digestion of pAdTrack-CMV-miR-16 by 12 g/L agarose gel electrophoresis. Lane 1, pAdTrack-CMV digested by *Kp*n I plus *Xho* I; lane 2, pAdTrack-CMV-miR-16 digested by *Kpn* I plus *Xho* I; lane 3, PCR product of rat miR-16 precursor DNA; Lane 4, DL2000 DNA Ladder Marker. (*B*) Restriction endonuclease digestion of rAdTrack-miR-16 by electrophoresis through an 8 g/L agarose gel and ethidium bromide staining. Lane 1: pAdTrack-CMV digested by *Pac* Ⅰ; Lane 2: rAdTrack-miR-16 digested by *Pac*Ⅰ; Lane 3: pAdEasy-I digested by *Pac*I. (*C*) Packaging of rAd-miR-16 in HEK293 cells. Magnification ×200. (*D*) MiR-16 expression in NRVCs with infection of rAd-miR-16. MOI=5, (*n*=3).

**Figure 3. Cell cycle distribution of NRVCs.**

(*A*) Cell cycle distribution of PE-treated NRVCs as analyzed by flow cytometry (n=3). (*B*) Cell cycle distribution of miR-16-overexpressing NRVCs as analyzed by flow cytometry (n=3). Data are shown as mean±SD; **p*<0.05, ***p*<0.01, ****p*<0.001.
